# Diprenylated Indole Alkaloids from Fruits of *Hexalobus monopetalus*

**DOI:** 10.1007/s13659-014-0010-x

**Published:** 2014-04-02

**Authors:** Hamisi M. Malebo, Stephan A. Jonker, Reiner Waibel, Mayunga H. H. Nkunya

**Affiliations:** 1 Department of Traditional Medicine Research, National Institute for Medical Research, P.O. Box 9653, Dar es Salaam, Tanzania; 2 Department of Chemistry, University of Dar es Salaam, P.O. Box 35061, Dar es Salaam, Tanzania; 3 Department of Pharmaceutical Chemistry, Institute of Pharmacy, University of Erlangen, Schuhstrasse 19, 91052 Erlangen, Germany

**Keywords:** *Hexalobus monopetalus*, Hexalobines, Indole alkaloids, *Candida albicans*

## Abstract

New hexalobine type alkaloid, 5-(2″,3″-epoxy-3″-methylbutyl)-3-(3′-hydroxy-3′-methyl-1′-acetyloxy-but-2′-yl)indole (**1**) alongside the known hexalobines 3-(2′,3′-dihydroxy-3′-methylbutyl)-5-(3″-methylcrotonoyl) indole (**2**), 3,5-hexalobine C (**3**) and 3,5-hexalobine D (**4**) were isolated from fruits of *Hexalobus monopetalus*. Compounds **3** and **4** exhibited antifungal activity against *Candida albicans*.

## Introduction

*Hexalobus* is one of the genera of the family Annonaceae which is confined to the Savannah region of tropical Africa. It is a relatively small genus of about six species comprising of erect shrubs or small trees [1, 2]. Only *Hexalobus monopetalus* is known to occur in Tanzania, being found in Coast, Iringa, Kigoma, Mbeya, Mwanza, Ruvuma and Tabora regions where it is used for the treatment of stomach disorders and fevers [3].

**Fig. 1:**
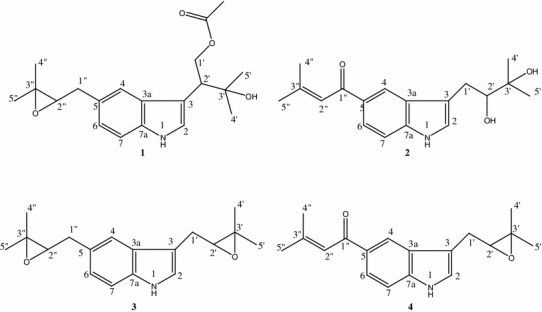
Structures of compounds **1**–**4**

The crude extracts from the root bark of *H. monopetalus* exhibited cytotoxicity in the brine shrimp lethality test with IC_50_ values ranging from 0.56 to 66.07 μg/mL [3]. In the antimalarial test, the crude ethanolic root bark extract was mildly active against the multidrug resistant K1 and chloroquine sensitive NF 54 strains of *Plasmodium falciparum*, with IC_50_ values of 9.9 and 13 μg/mL, respectively [3]. The same extract exhibited trypanocidal activity against *Trypanosoma brucei rhodesiense,* with a minimum inhibition concentration of 11 μg/mL [3]. These results prompted us to investigate crude extracts from the fruits of *H. monopetalus* for bioactive constituents. Furthermore, the fruits of *H. monopetalus* which are edible to primates and humans, were not hitherto investigated for their phytochemical constituents and hence were considered to be a target for these studies that formed part of long-term investigations of Annonaceae species occurring in Tanzania. This investigation resulted in the isolation of one new hexalobine type alkaloid compound **1** alongside the known hexalobines 3-(2′,3′-dihydroxy-3′-methylbutyl)-5-(3″-methylcrotonoyl) indole (**2**), 3,5-hexalobine C (**3**) and 3,5-hexalobine D (**4**) (Fig. 1). The structures of hexalobines were determined on the basis of spectroscopic analysis including NMR, MS, IR data and by comparison with data reported in literature [4–7].

## Results and Discussion

5-(2″,3″-Epoxy-3″-methylbutyl)-3-(3′-hydroxy-3′-methyl-1′-acetyloxybut-2′-yl)indole (**1**) was obtained as a colorless oil. The molecular formula C_20_H_27_NO_4_ was established on the basis of ^1^H, ^13^C NMR (Tables 1, 2), and EI-MS, and on comparison of the spectroscopic properties of hexalobines reported in the literature. The ^1^H NMR spectrum revealed the presence of five low field proton signals, which were attributed to a disubstituted indole skeleton [5, 6]. The broad singlet at *δ* 8.12 (1H) is typical for an indoyl N–H proton, the broadening of the signal arising from the quadrupole broadening effect of nitrogen [8]. The signal at *δ* 7.12 (1H, *J* = 2, H-2) appeared as a doublet due to coupling with an indoyl N–H, thus suggesting that C-3 on the indoyl moiety is substituted, as confirmed by the absence of an H-3 resonance at ca. *δ* 6.50 [7, 8]. Another low field signal appeared at *δ* 7.49 (1H, H-4) as a broad singlet and the presence of this signal indicated substitution at the indoyl position 5, as further revealed by the absence of an H-5 signal at ca. *δ* 6.99 [7, 8]. Two signals of *ortho*-coupled aromatic protons appeared at *δ* 7.34 (d, 1H, *J* = 8.0 Hz, H-7) and at δ 7.10 (dd, 1H, *J* = 8.0, 1.5 Hz, H-6) [4]. These indicated that the indole unit had a 1,2,4 arrangement of protons on the benzene ring [7].

**Table 1:** ^1^H NMR data of compounds **1**–**4**

H	*δ*_H_ J (Hz)			
	**1**	**2**	**3**	**4**
1	8.12 br. s	8.39 br. s	7.99 br. s	8.76 br. s
2	7.12 d (2)	7.17 d (2)	7.07 d (2.1)	7.11 d (2)
4	7.49 br. s	8.26 br. s	7.49 br. s	8.30 br. s
6	7.10 dd (8.0, 1.5)	7.85 dd (8.0, 1.5)	7.27 dd (8.1, 1.5)	7.86 dd (8.5, 1.5)
7	7.34 d (8.0)	7.37 d (8.5)	7.05 d (8.1)	7.35 d (8.5)
1′ a	4.52 dd (11.5, 7.5)	2.80 dd (14.5, 10.5)	3.06 m	3.03 m
1′ b	4.59 dd (11.5, 6.5)	3.09 dd (14.5, 2.5)	–	–
2′	3.41 dd (7.5, 6.5)	3.75 ddd (10.5, 2.5, 2.5)	2.93 m	3.09 m
4′	1.28^a^ s	1.33^a^ s	1.34^a^ s	1.36^a^ s
5′	1.31^a^ s	1.36^a^ s	1.44^a^ s	1.45^a^ s
1″ a	2.91 m	–	3.06 m	2.91 m
1″ b	2.9–3.1 m	–	–	2.9–3.1 m
2″	2.9–3.1 m	6.84 qq (1.5, 1.5)	2.93 m	6.86 m
4″	1.35 s	2.04 d (1.5)	1.35^b^ s	2.04^b^ d (1.5)
5″	1.44 s	2.21 d (1.5)	1.44^b^ s	2.20^b^ d (1.5)
OH	1.82 br. s	2.17 br. d (2.5)	–	–
OH	–	2.26 br. s	–	–
–OCOCH_3_	1.95 s	–	–	–

^a,b^Signals interchangeable within a column

**Table 2:** ^13^C NMR data of compounds **1**–**4**

C	*δ*_C_			
	**1**	**2**	**3**	**4**
2	122.6, CH	124.0, CH	122.11, CH	123.2, CH
3	113.5, C	114.5, C	112.27, C	114.0, C
3a	128.6, C	127.2, C	127.83, C	127.1, C
4	119.2, CH	122.0, CH	118.62, CH	120.9, CH
5	129.3, C	131.5, C	129.02, C	131.0, C
6	123.3, CH	120.6, CH	123.26, CH	122.7, CH
7	111.2, CH	111.0, CH	111.20, CH	111.0, CH
7a	134.8, C	139.0, C	135.19, C	138.5, C
1′	65.2, CH_2_	28.0, CH_2_	24.90, CH_2_	25.1, CH_2_
2′	46.2, CH	78.0, CH	64.11, CH	64.0, CH
3′	72.7, C	73.0, C	58.69, C	58.6, C
4′	28.3, CH_3_	26.5, CH_3_	24.86, CH_3_	24.9, CH_3_
5′	28.0, CH_3_	23.9, CH_3_	18.72, CH_3_	19.0, CH_3_
1″	35.5, CH_2_	193.0, C=O	35.53, CH_2_	192.3, C=O
2″	65.1, CH	123.0, CH	65.19, CH	122.4, CH
3″	58.7, C	154.0, C	58.69, C	154.0, C
4″	24.9, CH_3_	27.0, CH_3_	24.86, CH_3_	27.4, CH_3_
5″	19.0, CH_3_	20.9, CH_3_	18.88, CH_3_	21.0, CH_3_
–CH_3_	29.7	–	–	–
–C=O	171.1	–	–	–

The ^13^C NMR spectrum (Table 2) confirmed the presence of the 3,5-disubstituted indole nucleus as the methine C signals which normally resonate at ca. *δ* 102.10 for C-3 and at ca. *δ* 121.7 for C-5 in a typical unsubstituted indole alkaloid [7], now appeared as quaternary C signals at *δ* 113.5 and *δ* 129.3, respectively. Other indole C signals appeared in the anticipated chemical shifts typical for the indole nucleus (Table 2).

An ABX spin system was observed for **1**, with signals at *δ*_HA_ = 4.52 (H-1′*α*), δ_HB_ = 4.59 (H-1′*β*) and *δ*_HX_ = 3.41 (H-2′, *J*_AB_ = 11.5, *J*_AX_ = 7.5, *J*_BX_ = 6.5 Hz). The low field position of the AB methylene signals suggested that the methylene group in **1** was not directly connected to the indole moiety as in **2**, but with clear difference due to a stronger deshielding substituent which, based on the presence of a carbonyl absorption at *δ* 171.1 in the ^13^C NMR spectrum and a methyl H absorption at *δ* 1.95, was concluded to be an acetoxy group. The existence of the downfield ^1^H NMR resonances of 1′*α* at *δ*4.52 (dd, 11.5, 7.5) and 1′*β* at *δ*4.59 (dd, 11.5, 6.5) as compared with other hexalobines, confirms that electron-withdrawing group is nearby the germinal protons and the only possibility is the acetoxy group. Further analysis of the ^1^H NMR spectroscopic data confirmed the diastereotopic protons 1′*α* and 1′*β* are coupling with the methine resonating at *δ*3.41 (dd, 7.5, 6.5, H-2′). The prenyl linkage indicated by ^1^H NMR involving the acetoxy group, the diastereotopic protons and the methine led to only one structural possibility for **1**. While all the functional groups are well accommodated for in this structure, any other structural placement of the acetoxy group lacks support of ^1^H NMR spectroscopic data. Fortunately, there exist no any possible spectroscopic ambiguities in the placement of the acetoxy group in position 1′.

**Scheme 1:**
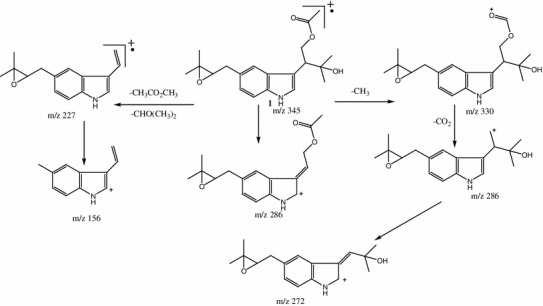
Mass spectral fragmentation pattern of 5-(2″,3″-epoxy-3″-methylbutyl)-3-(3′-hydroxy-3′-methyl-1′-acetyloxybut-2′-yl)indole (**1**)

The MS of **1** exhibited the M^+^ peak at *m*/*z* 345 corresponding to a molecular formula C_20_H_27_NO_4_. The odd mass of the EI-MS base peak at *m*/*z* 227 (C_15_H_17_NO^+^) implied its formation being through a rearrangement reaction, which is feasible due to the presence of the acyl substituent in **1** (Scheme 1). Loss of the epoxyisobutylene group from the fragment ion at *m*/*z* 227 ([C_15_H_17_NO]^+^) could explain the formation of the fragment ion at *m*/*z* 156 ([C_11_H_10_N]^+^). The subsequent loss from the molecular ion of a methyl radical and CO_2_, as indicated by the appearance of fragment ions at *m*/*z* 330 ([C_19_H_24_NO_3_]^+^) and 286 ([C_18_H_24_NO_2_]^+^) respectively, confirmed the presence of an acetyl group in structure **1**.

Compound **1** is hitherto not been reported but the presence of an acetyl group in this prenyl indole immediately led to the suspicion that the compound might be an artefact, being formed through transesterification of the corresponding hydroxy compound with acidic ethyl acetate used in the isolation process. However, it can also be argued that acidic conditions favouring transesterification with ethyl acetate during the isolation process would invariably also open the epoxide ring on the C-5 prenyl group. Apparently, a diol of **1** thought to be formed this way was not obtained when **1** was re-subjected to the isolation conditions using the same ethyl acetate as before. Furthermore, compound **1** was already detected in the crude extract which had hitherto not been subjected to chromatography in ethyl acetate. It is therefore concluded that **1** is a genuine natural product and not an artefact. Similar acyl bisprenylindoles have previously been isolated from *H*. *crispiflorus* [1, 3]. Biogenetically, hexalobines with an ester functionality like other hexalobines are conceived to be biosynthesised in these plants from tryptophan, and two C_5_ isoprene units from the isoprene pathway to form diprenyl indole skeleton (see Scheme 1.1) [1].

Compounds **3** and **4** exhibited very mild antifungal activity against *Candida albicans*, with zones of inhibition of 5 and 14 mm at a concentration of 100 μg/mL, respectively. In the previous investigations compound **4** exhibited antifungal activity against *Botrytis cinerea*, *Rhizoctonia solani* and *Saprolegnia asterophora* with zones of inhibition of 5, 5–12 and 5–12 at a concentration of 150 μg/mL [4].

## Experimental Section

### General Experimental Procedures

Infrared (IR) spectra, taken in chloroform solutions were recorded on a Shimadzu Model IR-435 spectrophotometer with absorptions given in wave numbers (cm^−1^). ^1^H NMR spectra were recorded on either a Bruker AM 360 instrument operating at 360 MHz with CDCl_3_ was used as solvent at the Institute of Pharmacy, University of Erlangen in Germany. Column chromatography was carried out with silica gel (200–300 mesh) and Sephadex LH-20 (Amersham Biosciences, Sweden). Fractions were monitored by thin layer chromatography (TLC). Visualization of TLC spots was done under UV light at 254 or 366 nm and by spraying with an anisaldehyde reagent. Detection was done under UV light at 254 or 366 nm. Vacuum liquid chromatography (VLC) was carried out using normal phase silica gel [of particle size 400 Mesh ASTM (Merck)] and gradient elution was applied. The vacuum was generated from a membrane pump.

### Plant Materials

The fruits of *H*. *monopetalus* were collected from Ugowola village in Tabora District in Tanzania. Identification of the plant species was done at the Herbarium of the Department of Botany, University of Dar es Salaam where a voucher specimen has been deposited (Manoko collection No. 0046-98).

### Extraction and Isolation of Compounds

The air dried and pulverised fruits of *H*. *monopetalus* (1 kg) were soaked in ethanol for 48 h at room temperature (about 30 °C). The crude extract was obtained by filtration, followed by concentration of the filtrate in vacuo using a rotary evaporator maintained at 30 °C so as to avoid decomposition of thermally unstable compounds. The resulting extract weighed 30 g. The crude extract was fractionated by VLC eluting with pet ether containing increasing amounts of ethyl acetate and then a mixture of methanol and ethyl acetate. The first five fractions contained a complex mixture of non-polar compounds and therefore they were not analyzed further. The combined 6th to 10th VLC fractions were separated by flash column chromatography on silica gel eluting with a 3:7 (v/v) mixture of ethyl acetate and pet ether, then gradually increasing the gradient to 1:1 (v/v) and finally with only ethyl acetate. This yielded 72 fractions which were combined to 13 based on TLC similarities. On repeated column chromatography of VLC fraction 11, 12 and 13 led to the isolation of hexalobines which were purified by chromatography on Sephadex^®^ LH-20 (MeOH).

### Antifungal Tests

The antifungal assay to evaluate the ability of the pure compounds to inhibit growth of *C*. *albicans* in a culture media was carried out using the standard plate diffusion method. The medium was prepared as follows: 32.50 g of Sabouraud Dextrose Agar was mixed with 500 mL of sterile distilled water. The mixture was sterilized by autoclaving at 120 °C for 15 min under 1 bar pressure. Under aseptic conditions in the laminar flow hood, the medium was dispensed into 150 mm pre-sterilized petri dishes to yield a uniform depth of 4 mm. They were then covered and allowed to cool and hardened at room temperature. The hardened medium was inverted and then incubated at 37 °C for the sterility assurance test. The microbial nutrient broth (2 g) was mixed with 250 mL of sterile distilled water. The mixture was sterilized by autoclaving at 120 °C for 15 min under 1 bar pressure. The nutrient broth was cooled, and an innoculum from a pure subculture of a *C*. *albicans* colon was innoculated into the broth and then diluted threefold, then introduced into the culture medium. Four circular wells were made in each culture medium and 10 μL containing 100 μg/mL of pure compounds dissolved in dimethyl sulfoxide (DMSO) was dispensed into each of the three wells in the medium, the fourth one being dispensed with 10 μL of DMSO, as a control. After the compounds had diffused into the medium, the culture medium was inverted and incubated at 37 °C for 24 h. The absence of a clear circular region around the disc loaded with a measured volume of test compound was used as an indicator of growth. The inhibition zone was determined by measuring the diameter in millimetres of the circular region around each well.

### 5-(2″,3″-Epoxy-3″-methylbutyl)-3-(3′-hydroxy-3′-methyl-1′acetyloxy-but-2′-yl)indole (**1**)

Yield: 10.2 mg. Anisaldehyde: brown. Cerium reagent: yellow. MS, *m*/*z* (% rel. int.) 345 (M^+^, 5), 330 (5), 285 (8), 270 (8), 244 (5), 227 (100), 212 (40), 198 (25), 184 (38), 172 (40), 156 (100), 143 (38), 115 (18), 83 (15), 59 (38) and 43 (60). ^1^H and ^13^C NMR: see Tables 1 and 2.

### 3-(2′,3′-Dihydroxy-3′-methylbutyl)-5-(3″-methylcrotonoyl)indole (**2**)

Colourless oil. Yield: 8.2 mg. Anisaldehyde: brown. Cerium reagent: orange. IR ν_max_ 3655, 3540, 1664, 1598 and 1573 cm^−1^. MS, *m*/*z* (% rel. int.) 301 (M^+^, 50), 286 (10), 243 (4), 212 (68), 198 (30), 130 (42), 83 (100), 59 (22) and 55 (23). ^1^H and ^13^C NMR: see Tables 1 and 2.

### 3,5-Hexalobine C (**3**)

Colourless oil. Yield: 9.1 mg. Anisaldehyde: brown. Cerium reagent: red. IR ν_max_ 3464, 2991 and 1630 cm^−1^. MS, *m*/*z* (% rel. int.) 285 (M^+^, 98), 270 (60), 242 (98), 214 (58), 156 (50) and 143 (100). ^1^H and ^13^C NMR: see Tables 1 and 2.

### 3,5-Hexalobine D (**4**)

White needles. Yield: 150 mg, m.p 131–133 °C. Anisaldehyde: yellow. Cerium reagent: brown. IR ν_max_ 3452, 2991, 1644, 1614 and 1571 cm^−1^. MS, *m*/*z* (% rel. int.) 283 (M^+^, 50), 268 (30), 240 (38), 228 (38), 210 (60), 196 (43), 129 (50) and 83 (100). ^1^H and ^13^C NMR: see Tables 1 and 2.
